# Supplementary material for: Comparison of blood viscosity models in different degrees of carotid artery stenosis
Source: PeerJ. 2025 Apr 28;13:e19336. doi: 10.7717/peerj.19336 (PMC12045283; doi:10.7717/peerj.19336)
Supplement: Supplemental Information 2 [file peerj-13-19336-s002.docx]

**3D models**

The 3D models used in this study are available at MorphoSource: <https://doi.org/10.17602/M2/M683636>；

<https://doi.org/10.17602/M2/M683642>；<https://doi.org/10.17602/M2/M683648>；

<https://doi.org/10.17602/M2/M683654>；<https://doi.org/10.17602/M2/M683660>；

<https://doi.org/10.17602/M2/M683666>；<https://doi.org/10.17602/M2/M683672>；

<https://doi.org/10.17602/M2/M683677>；<https://doi.org/10.17602/M2/M683685>；

<https://doi.org/10.17602/M2/M683691>；<https://doi.org/10.17602/M2/M683698>；

<https://doi.org/10.17602/M2/M683705>；<https://doi.org/10.17602/M2/M683711>；

<https://doi.org/10.17602/M2/M683717>；<https://doi.org/10.17602/M2/M683723>；

<https://doi.org/10.17602/M2/M683729>；<https://doi.org/10.17602/M2/M683735>；

<https://doi.org/10.17602/M2/M683741>；<https://doi.org/10.17602/M2/M683747>；

<https://doi.org/10.17602/M2/M683753>；<https://doi.org/10.17602/M2/M683759>；

<https://doi.org/10.17602/M2/M683766>；<https://doi.org/10.17602/M2/M683771>；

<https://doi.org/10.17602/M2/M683786>；<https://doi.org/10.17602/M2/M683792>；

<https://doi.org/10.17602/M2/M683798>；<https://doi.org/10.17602/M2/M683804>；

<https://doi.org/10.17602/M2/M683810>；<https://doi.org/10.17602/M2/M683816>；

<https://doi.org/10.17602/M2/M683822>；<https://doi.org/10.17602/M2/M683828>；

<https://doi.org/10.17602/M2/M683834>；<https://doi.org/10.17602/M2/M683840>；

**Measurement of Hemodynamic Metrics**

The WSS at the stenosis during the extraction of peak blood flow velocity in the third cardiac cycle is represented by $\vec{\tau}_{wss}$. T is the cardiac cycle. The equations for the parameters TAWSS, OSI, and RRT based on WSS can be expressed as follows:

| $\mathrm{TAWSS}=\frac{1}{T}\int_{0}^{T} \left\vert\vec{\tau}_{wss} \right\vert dt$ | (1) |
| --- | --- |
| $\mathrm{OSI}=\frac{1}{2}\left( 1-\left\vert\int_{0}^{T} \vec{\tau}_{wss}dt \right\vert/{\int_{0}^{T} \left\vert\vec{\tau}_{wss} \right\vert}dt \right)$ | (2) |
| ${\mathrm{RRT}=1}/{\left( 1-2\mathrm{OSI} \right)\mathrm{TAWSS}}$ | (3) |
